# Impact of a monthly antimicrobial stewardship quality assurance tool for elevated vancomycin levels

**DOI:** 10.1017/ash.2022.376

**Published:** 2023-03-15

**Authors:** Olivia C. Knack, Alice M. Landayan, Kelsey N. Williams, Lee M. Amaya, Timothy P. Gauthier

**Affiliations:** 1 Clinical Pharmacy Enterprise, Baptist Health South Florida, Miami, Florida; 2 Department of Pharmacy, Baptist Health South Miami Hospital, Miami, Florida; 3 Department of Pharmacy, Baptist Hospital of Miami, Miami, Florida; 4 Department of Pharmacy, Baptist Health Miami Cancer Institute, Miami, Florida

## Abstract

**Objective:**

We sought to determine the value of an audit-and-feedback monitoring method in facilitating meaningful practice changes to improve vancomycin dosing and monitoring.

**Design:**

Retrospective, multicenter, before-and-after implementation observational quality assurance initiative.

**Setting:**

The study was conducted in 7 not-for-profit, acute-care hospitals within a health system in southern Florida.

**Methods:**

The preimplementation period (September 1, 2019, through August 31, 2020) was compared to the postimplementation period (September 1, 2020, through May 31, 2022). All vancomycin serum-level results were screened for inclusion. The primary end point was the rate of fallout, defined as vancomycin serum level ≥25 µg/mL with acute kidney injury (AKI) and off-protocol dosing and monitoring. Secondary end points included the rate of fallout with respect to AKI severity, rate of vancomycin serum levels ≥25 µg/mL, and average number of serum-level evaluations per unique vancomycin patient.

**Results:**

In total, 27,611 vancomycin levels were analyzed from 13,910 unique patients. There were 2,209 vancomycin serum levels ≥25 µg/mL (8%) among 1,652 unique patients (11.9%). AKI was identified in 379 unique patients (23%) with a vancomycin levels ≥25 µg/mL. In total, 60 fallouts (35.2%) occurred in the 12-month preimplementation period (∼5 per month) and 41 fallouts (19.6%) occurred in the 21-month postimplementation period (∼2 per month; *P* = .0006). Failure was the most common AKI severity in both periods (risk: 35% vs 24.3%, *P* = .25; injury: 28.3% vs 19.5%, *P* = .30; failure: 36.7% vs 56%, *P* = .053). Overall, the number of evaluations of vancomycin serum levels per unique patient remained consistent throughout both periods (2 vs 2; *P* = .53).

**Conclusions:**

Implementation of a monthly quality assurance tool for elevated outlier vancomycin levels can improve dosing and monitoring practices resulting in enhanced patient safety.

Intravenous (IV) vancomycin is commonly prescribed for the empiric or targeted treatment of infections caused by gram-positive bacteria, such as methicillin-resistant *Staphylococcus aureus*. IV vancomycin requires therapeutic drug monitoring (TDM) to balance between safety and efficacy. This balance is commonly accomplished through pharmacy-to-dose (PTD) protocols, wherein pharmacists are given the authority to order laboratory tests and adjust doses to achieve goal levels. Despite close monitoring, acute kidney injury (AKI) may occur during IV vancomycin therapy. This requires action to avoid drug accumulation, which may in turn contribute to further worsening renal function.^1^ Nonmodifiable risk factors for vancomycin-associated AKI include primary history of kidney disease, obesity, intensive-care unit admission, and age ≥65 years.^2^ Vancomycin-specific risk factors that may contribute to AKI development include trough levels above 20 µg/mL, total daily doses of 4,000 mg or more, therapy durations exceeding 7 days, and concomitant nephrotoxic agents.^3^ Although elevated vancomycin levels may be secondary to AKI rather than the cause, investigation of such outlier levels can reveal opportunities for improvement in dosing and monitoring practices.

Intravenous (IV) vancomycin is one of the most commonly prescribed antibiotics in the acute-care setting, listed as one of 10 most prescribed antibiotics from 2010 to 2015.^4^ The Centers for Disease Control and Prevention (CDC) cited a 32% increase in vancomycin use across hospitals nationwide from 2006 to 2012,^5^ with one projection estimating a 200% usage increase by 2030.^6^ High utilization and the risk for toxicity makes IV vancomycin a priority item and common target for antimicrobial stewardship programs (ASPs). Support via standardized practice auditing tools and continuous quality-assurance measures will be crucial for addressing these high-priority targets.

To identify and address opportunities related to the dosing and monitoring of this high-priority target, an auditing tool was implemented to be completed retrospectively on a recurring monthly basis. The purpose of this project was to assess the impact of the antimicrobial stewardship quality-assurance monitor for IV vancomycin in facilitating meaningful practice changes.

## Methods

This was a retrospective multicenter evaluation of a new quality-assurance initiative implemented across 7 hospitals in southern Florida. The intervention, a monthly retrospective audit with action and feedback, was added to the existing PTD program. The vancomycin dosing and monitoring guidelines and practices for routine evaluation of appropriateness were unmodified throughout the study period. The preimplementation period of September 1, 2019, through August 31, 2020 (12 months) was compared to the postimplementation period of September 1, 2020, through May 31, 2022 (21 months). All patients with a vancomycin serum-level results were included. Patients receiving hemodialysis prior to vancomycin initiation were excluded from the AKI-related outcomes assessment. This evaluation was exempt from institutional review board review.

The primary outcome of this initiative was the rate of fallout, defined as AKI with level ≥ 25 µg/mL and dosing and/or monitoring that did not follow protocol. Identified fallouts were categorized based on fallout reason. Definitions are provided in Table 1.

**Table 1.** Definition for Fallout Reason With Example

Reason for Fallout	Definition
Late monitoring	Vancomycin level obtained later than recommended on institution protocol
Therapeutic prior to steady state—dose continued	Prespecified goal level achieved prior to steady state and necessary dose and monitoring adjustment not made
Redosed after vancomycin serum level was >20 µg/mL	Vancomycin re-dosing too soon following elevated outlier result
Lack of consideration for patient-specific data	Patient specific factors not taken into consideration in dosing and monitoring plan (eg, serum creatinine, urine output, weight, age)
Lack of consideration for historical vancomycin data	Vancomycin-specific historical data available but not taken into consideration leading to inappropriate dosing and monitoring plan
Dose and frequency changed	Both dose and frequency changed but per protocol only dose or frequency should have been changed
Received intraoperative dose	Vancomycin dose administered intraoperatively and not accounted for in postoperative dosing and monitoring plan
Maintenance dose increased without level	Vancomycin maintenance dose increased in absence of vancomycin level

Secondary outcomes included rate of fallout with respect to AKI severity per risk, injury, failure, loss, and end-stage kidney disease (RIFLE) criteria, rate of vancomycin serum levels ≥25 µg/mL, and vancomycin serum-level evaluations per unique vancomycin patient. AKI severity classification was limited to risk (defined as serum creatinine level 1.5–1.9 times baseline or ≥0.3 mg/dL increase from baseline), injury (defined as serum creatinine level 2.0–2.9 times baseline), or failure (defined as serum creatinine level 3.0 times baseline or ≥ 4.0 mg/dL with an acute rise of >0.5 mg/dL).^7^

**Fig. 1.**
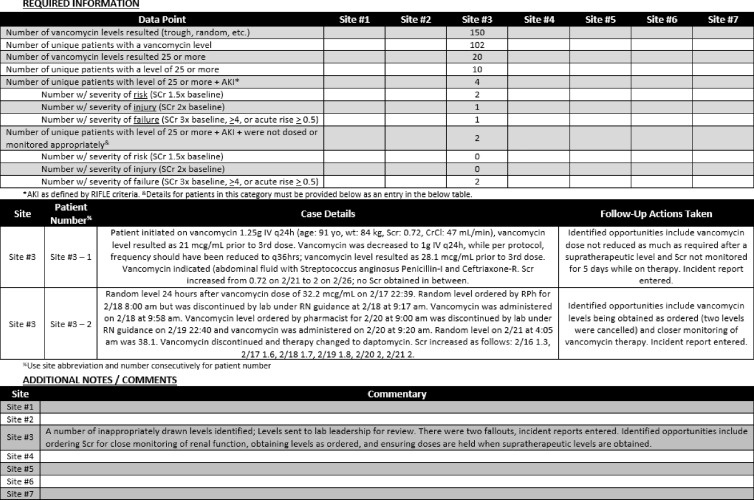
Sample vancomycin monitor.

This vancomycin monitoring method directs audits of all vancomycin serum levels ≥25 µg/mL on a recurring monthly basis using a stepwise approach with the overall intention of streamlining for efficiency (Fig. 1). All vancomycin serum levels are captured in a report from Cerner Millennium Discern Analytics version 2.0 software (Cerner, Kansas City, MO). Vancomycin serum levels ≥25 µg/mL are identified from the report via filters in Microsoft Excel (Microsoft, Redmond, WA). The antimicrobial stewardship pharmacist reviews each unique patient chart with a vancomycin serum level ≥25 µg/mL, assesses baseline renal function, and determines whether AKI occurred during vancomycin therapy. If AKI has occurred during vancomycin therapy, the severity of AKI is classified based on the Kidney Disease Improving Global Outcomes (KDIGO) RIFLE criteria, and further assessment is required to determine whether vancomycin dosing and/or monitoring was inappropriate according to the health system’s vancomycin dosing and monitoring protocol.^7^

The system-wide goal was set at zero fallouts per month; therefore, any identified fallout required further assessment. Each month, fallout cases are summarized and appropriate follow-up action(s) are identified and performed or planned. Overall results are summarized monthly at the system level. Frontline staff and leadership within laboratory, medicine, pharmacy, nursing, and elsewhere are engaged to varying degrees depending on each sites’ identified opportunity areas. Feedback regarding opportunities is communicated via e-mail, telephone, and/or in person. Additionally, educational 1-page flyers have been created and distributed to educate staff on common vancomycin dosing and/or monitoring pitfalls including dosing in the elderly, obesity, and unstable renal function.

A time commitment for the monitor was estimated by the primary investigator based upon experience completing the task for all sites for the preimplementation period and through collecting feedback from ASP pharmacist champions for the postimplementation period.

### Statistical analysis

All categorical data for the preimplementation and postimplementation periods were analyzed using the χ^2^ test. Significance was defined as *P* < .05, and all statistical analyses were performed using VassarStats software.

## Results

Throughout the study period, 27,611 vancomycin serum levels were assessed in 13,910 unique patients. In total, 2,209 vancomycin serum levels (8%) were ≥25 µg/mL, which occurred in 1,652 unique patients (11.9%). In the 12-month preimplementation period, 1,614 (12.7%) of 4,825 patients had a vancomycin serum level ≥25 µg/mL. In the 21-month postimplementation period, 1,038 (11.4%) of 9,085 patients had a vancomycin serum level ≥25 µg/mL (*P* = .024).

**Fig. 2.**
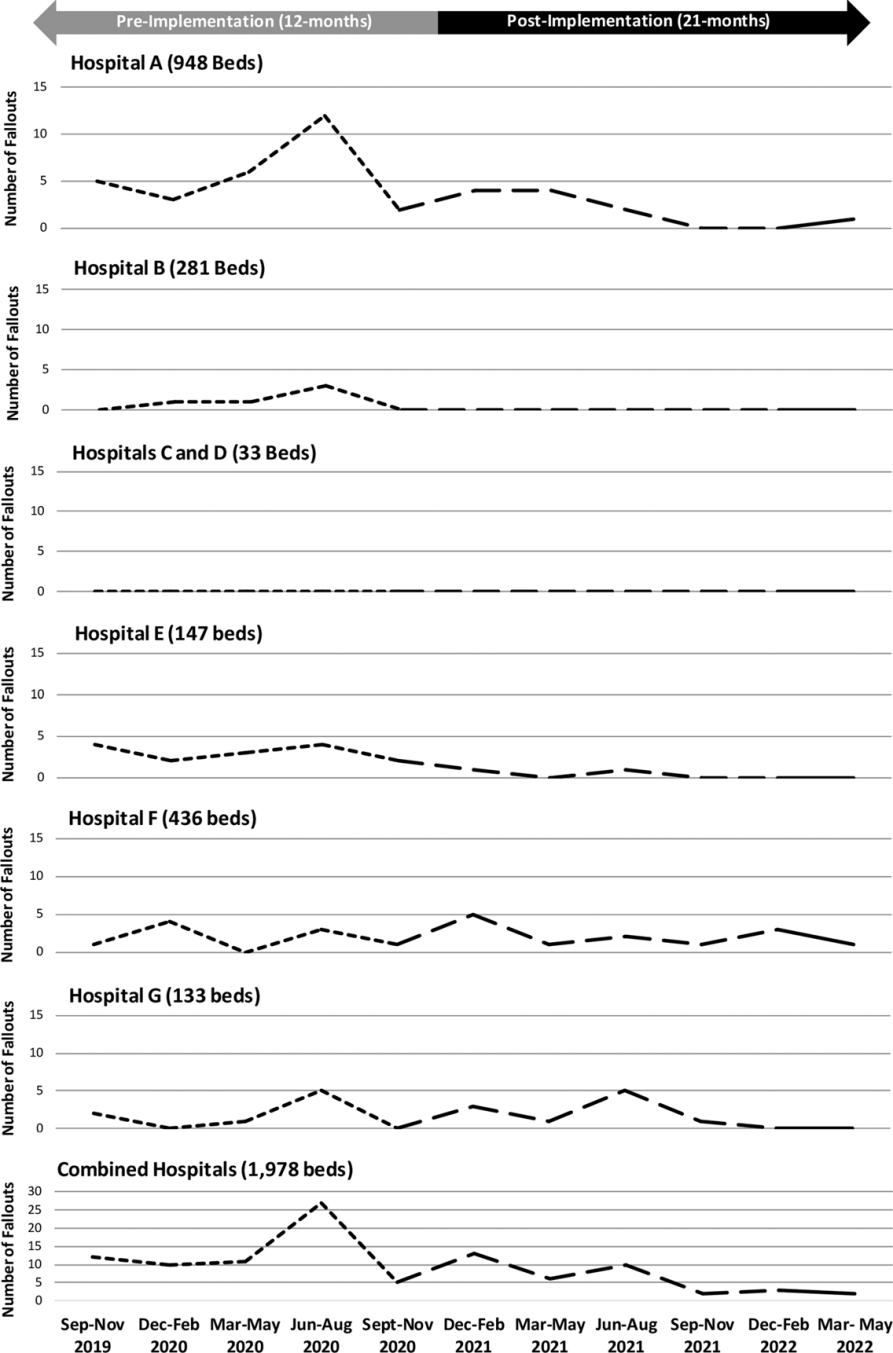
Vancomycin fall-outs per 3-month interval by site and combined.

**Fig. 3.**
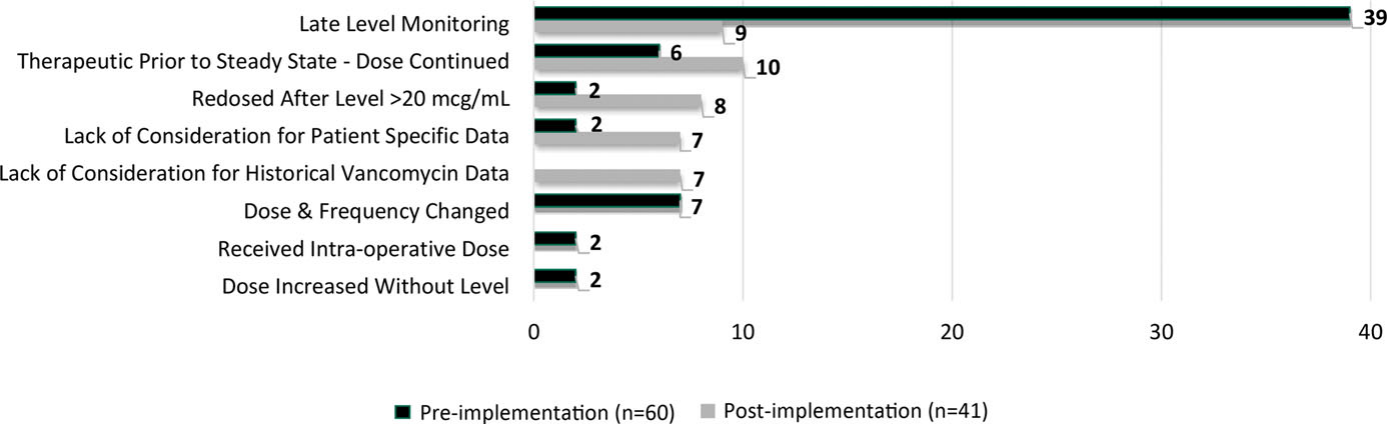
Determined reason for fallout by implementation phase.

In the cases in whom a vancomycin serum level was ≥25 µg/mL, 379 incidents of AKI were identified: 278 (73%) without fallout and 101 (27%) with fallout. In the 12-month preimplementation period, 614 unique patients had a vancomycin serum level ≥ 25 µg/mL, of whom 170 (27.7%) had AKI. In the 21-month postimplementation period, 1,038 unique patients had a vancomycin serum level ≥25 µg/mL, of whom 209 (20.1%) had AKI (*P* = .0004). The rates of AKI with fallout were 35.2% (n = 60) and 19.6% (n = 41) in the preintervention and postintervention periods, respectively (*P* = .0006). Figure 2 depicts fallout rates by institution and cumulatively in 3-month intervals across the study periods. The incidence of fallout decreased in the postimplementation period, with 2 fallouts in the most recent data point. Fallout designations comparing the study periods are provided in Figure 3.

When examining severity of AKI in patients with fallouts in the preintervention versus postintervention periods, the rates were 35% (n = 21) versus 24.3% (n = 10) for risk (*P* = .25), 28.3% (n = 17) versus 19.5% (n = 8) for injury (*P* = 0.3), and 36.7% (n = 22) versus 56% (n = 23) for failure (*P* = .053), respectively. Failure was the most common AKI severity for fallouts in the last 9 months of the postimplementation period (2 risk, 2 injury, 3 failure).

In the preimplementation period, 9,627 vancomycin serum levels were obtained for 4,825 unique patients and in the postimplementation period, 17,984 levels were obtained for 9,085 unique patients, averaging 2 vancomycin serum-level evaluations per unique patient for both periods (*P* = .53).

The time commitment to complete vancomycin monitoring was estimated at 2 minutes to screen a patient for AKI during vancomycin therapy and 10 minutes per fallout patient workup. Within the most recent month, 53 (13.5%) of 394 patients had a vancomycin serum level ≥25 µg/mL and needed to be screened for AKI. Among those 53 patients, only 10 (19%) were identified to have AKI during vancomycin therapy, and only 1 patient (10%) experienced fallout. The total time estimated to perform this assessment is 116 minutes (106 minutes to screen 53 patients for AKI plus 10 minutes for 1 fallout workup). Additional time was needed to review the report and fill out the form, but this was minimal. The time commitment for activities related to follow-up of fallouts and opportunities could not be quantified due to variability from site to site and over time, based upon institution-specific needs.

## Discussion

Intravenous vancomycin is a high-priority item and opportunity area due to the level of utilization within health systems and risk of nephrotoxicity to patients. Standardized tools for targeting IV vancomycin are necessary for ASP efforts to improve high-priority items. Implementation of a recurring monthly antimicrobial stewardship quality-assurance monitoring method for IV vancomycin contributed to improvements in dosing and monitoring practices, enhanced patient safety, and promoted staff accountability.

Over time, a decrease in fallouts was observed individually and cumulatively. As anticipated, achieving lower fallout rates was not immediate because it takes time for monthly case evaluation to spotlight opportunity areas and evoke action. Close monitoring is necessary for fallout prevention given the greater observed prevalence of AKI resulting in failure when a fallout did occur. We expected greater scrutiny to lead to earlier monitoring and increased levels overall. However, the number of average levels obtained per unique patient remained consistent throughout the initiative. Although not assessed in our initiative, based on historical experience, we suspect that the lack of increased vancomycin serum levels may have been due to short treatment durations.

Ultimately, it is important to recognize the multitude of contributing factors in AKI development. Therefore, complete avoidance of AKI during vancomycin therapy is unlikely, despite reasonable TDM practices. Continuous utilization and implementation of the system-level changes that result from the implementation of this monitoring method will be vital in ensuring a sustained reduction in fallouts.

The required time dedicated to performing this monthly monitoring was not substantial overall, offering a low-effort–high-payoff initiative to improve dosing and monitoring practices applicable to institutions of various sizes. Furthermore, we expect the time dedicated to decline over time as practice enhancements lead to a decrease in fallouts and system-level changes reinforce these practices. Time for follow-up on fallouts and other identified opportunities can be expected to vary considerably. Given that vancomycin optimization goes beyond acting on fallouts, a bundled approach to process improvement may be considered. This approach showed benefits in an observational before-and-after implementation cohort study that assessed a multi-interventional approach to improve vancomycin dosing and monitoring. Their intervention led to a lower incidence of vancomycin serum levels >20 µg/mL and reduced nephrotoxicity.^8^ Our vancomycin monitoring method was not designed to assess which actions should be performed in response to a specified fallout reason, but instead categorized reasons for fallout to allow identification of areas likely to need improvement within other institutions. Tailoring strategies to address fallouts according to identified opportunities is preferred over a universal approach, which may be infeasible.

Detecting ways to further enhance this monitoring method are necessary for its continued utilization and success. Two areas of opportunity are targeting vancomycin appropriateness and early vancomycin de-escalation. Monitoring IV vancomycin has many associated indirect costs (eg, ordering, drawing, processing, and interpreting levels).^9^ In the past, other vancomycin alternatives have proven to be cost-prohibitive, with the average daily cost of daptomycin (range, $450–750) and linezolid (range, $370–550) compared to vancomycin (range, $15–55).^10^ However, now that these alternative therapies are available as generic drugs, the continued first-line use of vancomycin for gram-positive infections in place of an alternative requiring little to no monitoring should be carefully considered.^11^

Our study had several limitations. One potential confounder was the onboarding of several antimicrobial stewardship pharmacists during the postimplementation period. Onboarding a new monitoring method within a health system and acclimation to the workflow is not a rapid process, and IV vancomycin optimization is only one of many items on an ASP pharmacist’s task list. The addition of several antimicrobial stewardship pharmacists certainly provided support to pharmacy staff and the initiative overall, but these pharmacists are not tasked with vancomycin dosing and monitoring, and their presence alone could not be expected to lead to the improvements that occurred. Another consideration to this work is that PTD vancomycin was not universal in the preimplementation period but was implemented systemwide during the postimplementation period. Even prior to this harmonization, several sites were already doing PTD on all patients, and the sites that went to universal PTD were already performing this service for most of their patients. Finally, this monitoring method was not designed or intended to assess appropriateness or indication of vancomycin therapy. An assessment of appropriateness would have been a valuable contribution; however, such prospective assessments are often infeasible at most institutions. The strength of the data is limited by the retrospective study design; however, this may have been counterbalanced by our use of objective end points.

In conclusion, improved dosing and monitoring practices of IV vancomycin revealed opportunities for targeted improvement efforts through interprofessional engagement and collaboration. Direct feedback to staff and leadership sparked conversations, created awareness, and shifted organizational culture surrounding antimicrobial stewardship within the health system. This monthly audit and feedback monitoring for elevated outlier vancomycin levels can be an effective tool for improving practices. Additionally, this monitoring method could serve as a continuous quality-assurance initiative to assess adherence to the health system’s vancomycin dosing and monitoring policy and to gauge the success of ASPs.
